# Comparative Analysis of Third Molar Segmentation Performance Across Dental Developmental Stages and the 18-Year Age Threshold Using Deep Learning Models

**DOI:** 10.3390/diagnostics16162636

**Published:** 2026-08-19

**Authors:** Melis Büşra Aşkın, Ayşe Bulut, Gökalp Çınarer

**Affiliations:** 1 Department of Orthodontics, Faculty of Dentistry, Yozgat Bozok University, 66100 Yozgat, Turkey; 2 Department of Oral and Maxillofacial Radiology, Physiology, Faculty of Dentistry, Yozgat Bozok University, 66100 Yozgat, Turkey; ayse.bulut@bozok.edu.tr; 3 Department of Computer Engineering, Faculty of Engineering-Architecture, Yozgat Bozok University, 66100 Yozgat, Turkey; gokalp.cinarer@bozok.edu.tr

**Keywords:** third molar, dental age estimation, 18-year threshold, Demirjian method, panoramic radiograph, YOLO, segmentation, forensic dentistry

## Abstract

**Background/Objectives:** Third molar development is one of the most frequently used dental indicators in forensic age assessment because its maturation continues through adolescence and early adulthood. Manual staging on panoramic radiographs requires experience and may be affected by observer variability, especially in transitional developmental stages. This study evaluated the performance of segmentation-based deep learning models for automatic third molar localization and Demirjian-based developmental stage classification on panoramic radiographs. **Methods:** The study used a two-stage segmentation framework. In the first stage, third molar localization was performed using 737 panoramic radiographs and 2736 annotations for teeth 18, 28, 38, and 48. In the second stage, developmental stage classification was performed using 695 panoramic radiographs and 2533 annotations grouped as AB, CD, EF, and GH according to Demirjian developmental stages. A supplementary 18-year threshold segmentation analysis was added using 695 panoramic radiographs and 2573 labels divided into training, validation, and test sets at an 80:10:10 ratio. YOLO-based segmentation models were trained using AdamW optimization and learning-rate settings recorded in the training logs. Model performance was evaluated using TP, FP, FN, precision, recall, F1-score, accuracy, mAP, Dice coefficient, Jaccard index, and performance curves. For instance segmentation, true negatives were not calculated because the number of background non-objects is not finite or clinically meaningful. **Results:** The third molar localization model yielded TP = 256, FP = 4, and FN = 11 on the test set. The overall accuracy was 0.9446, mAP@0.5 was 0.980, mAP@0.5:0.95 was 0.875, Dice coefficient was 0.9064, and Jaccard index was 0.8696. In developmental stage classification, yolo11x-seg produced the most balanced segmentation profile, with an accuracy of 0.7272, Dice coefficient of 0.5783, and Jaccard index of 0.5529. In the supplementary 18-year threshold segmentation analysis, yolov8x-seg produced TP = 172, FP = 37, and FN = 32. Overall accuracy was 0.8152, precision was 0.8230, recall was 0.8431, F1-score was 0.8329, mAP@0.5 was 0.635, and mAP@0.5:0.95 was 0.563. Class-wise results showed better performance for the under-18 class than for the 18-years-and-older class. **Conclusions:** YOLO-based segmentation models can localize third molars on panoramic radiographs with high performance. Developmental stage classification is more difficult than anatomical localization because the radiographic boundaries between adjacent developmental stages are gradual rather than discrete. The added 18-year threshold analysis provides a clinically relevant age threshold experiment, but it also shows that class imbalance and weak segmentation of the 18-years-and-older class limit direct forensic use. Segmentation-based third molar analysis should therefore be interpreted as a visually auditable decision-support workflow rather than a stand-alone legal age determination tool.

## 1. Introduction

Dental age estimation is widely used to assess biological development in forensic, clinical, and orthodontic settings. Tooth formation follows a relatively ordered developmental pattern and is less affected by many environmental factors than skeletal maturation. For this reason, mineralization-based dental indicators are often used in age estimation, growth monitoring, treatment planning, and human identification [1,2,3,4,5]. Reliable age assessment may become particularly important in cases involving unidentified individuals, missing birth records, migration, asylum procedures, criminal responsibility, or medico-legal age thresholds [3,6,7,8].

Panoramic radiography is one of the most frequently used imaging modalities for dental age estimation because it provides a single-view representation of the maxilla, mandible, developing teeth, root morphology, eruption status, and third molars. In radiographic age assessment, dental mineralization and root development are more stable indicators than eruption timing. Eruption may be influenced by crowding, impaction, local pathology, arch space, and environmental conditions, whereas mineralization stages provide a more structured view of dental maturation [1,2,9,10].

The Demirjian system remains one of the most established approaches for classifying dental development. It originally describes tooth maturation from stage A to stage H, based on crown formation, dentin deposition, root development, canal wall morphology, and apical closure [1,2]. Stages A and B represent early crown mineralization. Stages C and D describe crown formation and the first signs of root development. Stages E and F reflect progressive root formation, while stages G and H represent advanced root maturation and apical closure. Although the Demirjian method was first developed using mandibular permanent teeth, it has been adapted across different populations, age groups, and tooth types, including third molars [3,4,5,6,7,8,9,10,11,12,13].

Third molars have a special role in forensic age estimation. Most permanent teeth complete root development before late adolescence, whereas third molars continue to develop into adolescence and early adulthood. This makes them useful for evaluating legal age thresholds such as 14, 16, and 18 years [6,7,11,12,14]. However, third molar maturation is also variable. Agenesis, impaction, sex, jaw side, genetic background, population structure, and imaging quality may influence developmental timing and radiographic interpretation [6,7,8,9,10,11,12,13]. These factors explain why third molar-based age estimation requires population-specific validation and careful reporting of model performance.

Conventional dental age estimation relies on manual staging by trained observers. This process is time-consuming in large datasets and may be affected by intra-observer and inter-observer variability. Observer disagreement is more likely when developmental stages are close to each other, especially in transitional phases where crown and root morphology do not fit sharply into one category [3,5,10]. Grouping Demirjian stages as AB, CD, EF, and GH can simplify the developmental continuum into broader, clinically meaningful categories. Such grouping may also reduce the effect of small class size and support machine learning models by providing more stable class boundaries.

Artificial intelligence and deep learning methods have increasingly been used in dental radiographic analysis. These methods have been applied to tooth detection, tooth numbering, segmentation, periapical lesion detection, bone loss assessment, and dental age estimation [14,15,16,17,18,19,20,21,22,23,24,25,26]. Recent studies show that the field has moved from whole-image prediction toward tooth-level systems that can localize the relevant structure before assigning a label. Franco et al. reported AI-based binary classification of third molar development around the legal thresholds of 14, 16, and 18 years [14]. Milani et al. developed a fully automated model for lower third molar developmental staging [15]. Matthijs et al. validated an automated stage allocation system on mandibular tooth types [16], and Kokomoto et al. reported fully automatic dental age calculation from panoramic radiographs [17].

Additional recent studies support the value of structured tooth-level outputs. Ong et al. developed an automated Demirjian-based dental development staging system on panoramic radiographs [27]. Peker and Kurtoglu reported precision of 0.90, recall of 0.94, mAP50 of 0.968, and an F1-score of 0.919 for YOLOv10-based tooth detection and numbering in mixed dentition [28]. Beşer et al. evaluated YOLOv5 for detection, segmentation, and numbering of deciduous and permanent teeth in pediatric panoramic radiographs [29]. Putra et al. reported an accuracy of 88.5%, recall of 100%, and F1-score of 93.44% for automated permanent tooth detection and numbering [30]. These studies show that deep learning models can perform well in anatomical detection tasks, while developmental staging remains more sensitive to class definition, sample balance, and radiographic morphology.

AI-based dental age estimation generally follows two different approaches. In the first approach, the model predicts chronological age directly from the panoramic radiograph. In the second approach, teeth or specific dental regions are first detected, developmental stages are assigned, and age interpretation is performed through these intermediate outputs [15,16,17,18,19]. The second approach is more interpretable for clinical and forensic use because it shows the tooth and stage on which the decision is based. A visible intermediate output is easier to compare with expert assessment and may support auditability in medico-legal contexts.

YOLO-based models have become widely used in object detection and segmentation tasks because they provide fast inference and strong localization performance. In panoramic radiographs, segmentation is challenging because of anatomical overlap, variable contrast, impacted teeth, restorations, metal artifacts, superimposed structures, and differences in image quality. Segmentation models are useful in this setting because they assign a class and delineate the region used for the decision [24,25,26,28,29,30,31,32,,33]. In third molar analysis, automatic localization of teeth 18, 28, 38, and 48 can provide a controlled anatomical input for developmental stage classification. Third-molar-specific AI studies on impaction, surgical difficulty, and anatomical risk assessment also show that model performance is closely related to object visibility, angulation, overlap with neighboring structures, and the quality of the annotated reference standard [34,35,36].

The number of AI-based studies in dental age estimation is increasing, but studies that combine third molar localization and Demirjian-based developmental staging at segmentation level remain limited [14,15,16,17,18,19,20,21,22,23]. Many models estimate age directly from the whole radiograph or evaluate dental development without showing the exact segmentation region used for the decision. A two-stage segmentation approach may therefore provide a more transparent structure: first, the third molar is located; second, its developmental stage is classified. This design allows both the anatomical target and the developmental stage to be visually checked.

This study aimed to evaluate YOLO-based segmentation models for automatic third molar localization and developmental stage classification on panoramic radiographs. In the first stage, teeth 18, 28, 38, and 48 were localized using a segmentation model. In the second stage, third molar development was classified into AB, CD, EF, and GH groups based on Demirjian stages. The models were evaluated using accuracy, mAP, Dice coefficient, Jaccard index, precision–recall curves, F1–confidence curves, ROC curves, and confusion matrices.

The motivation for this work is based on the need for a visually interpretable and reproducible workflow for third-molar-based developmental assessment. Manual staging is useful but can be affected by observer variation. Whole-image age prediction may produce numerical output without showing which anatomical structures influenced the result. A segmentation-based model can provide a visible region of interest and a stage label at the same time.

The main contributions of this study are as follows:

1. A two-stage framework was constructed for third molar localization and developmental stage classification on panoramic radiographs.
2. Third molars were first segmented as 18, 28, 38, and 48 to evaluate anatomical localization performance.
3. Demirjian stages were grouped as AB, CD, EF, and GH and classified using segmentation-based YOLO models.
4. yolov8x-seg, yolo11x-seg, and yolo26x-seg were compared for developmental stage classification.
5. Model performance was evaluated using both detection-based and overlap-based metrics, including mAP, Dice coefficient, Jaccard index, confusion matrices, and performance curves.

A supplementary segmentation analysis was added for the legally relevant 18-year threshold, with binary classes defined as 18 years of age and older and under 18 years of age.

## 2. Materials and Methods

### 2.1. Study Design

This study used a two-stage deep learning segmentation design for third molar analysis on panoramic radiographs. The first stage focused on anatomical localization of third molars. The second stage focused on developmental stage classification based on grouped Demirjian stages. The localization task used four tooth-number classes: 18, 28, 38, and 48. The developmental stage task used four grouped stage classes: AB, CD, EF, and GH.

The AB group represented early development. The CD group represented advanced crown formation and early root formation. The EF group represented progressive root formation before complete apical closure. The GH group represented advanced root development with narrowing or closure of the apex.

**Figure 1:**
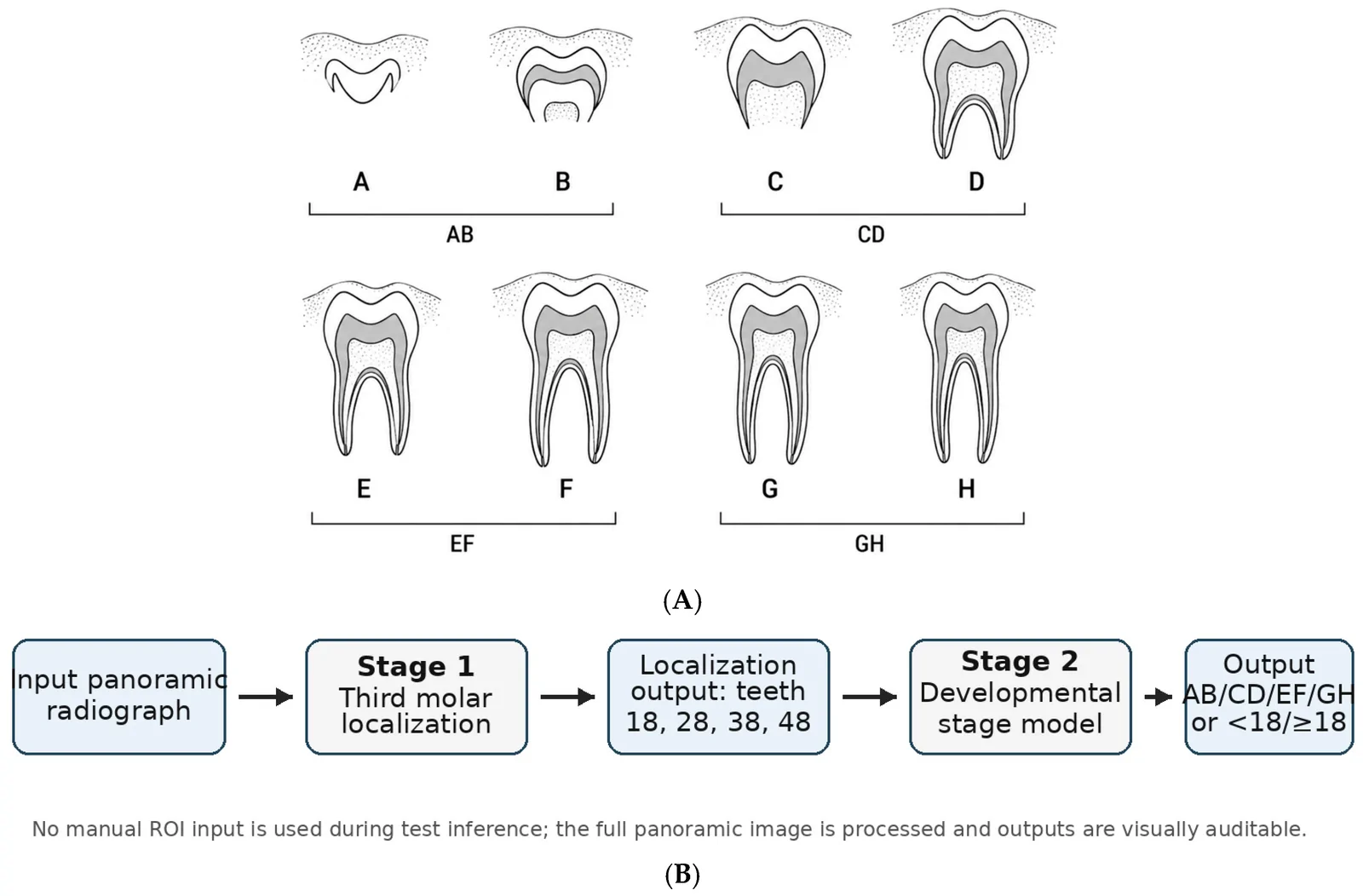
(**A**) Schematic representation of Demirjian developmental stages and the AB–CD–EF–GH grouping used in the present study. The illustration was redrawn by the authors based on the stage definitions of Demirjian et al. (**B**) Complete data flow and inference workflow of the segmentation-based third molar analysis. The full panoramic radiograph is processed first for third molar localization and then for developmental stage or age threshold segmentation output.

The data flow of the model was clarified as follows (Figure 1). During inference, the full panoramic radiograph is supplied to the third molar localization model. The first stage output identifies and segments the third molar regions corresponding to teeth 18, 28, 38, and 48. The developmental stage model then produces segmentation-based AB, CD, EF, or GH outputs. The workflow is therefore designed to provide both an anatomical target and a developmental label that can be visually checked by the examiner. No manual ROI input is required during test inference; the model output is generated from the radiograph and the trained segmentation weights. This distinction is important because the two-stage structure refers to a linked analytical workflow rather than to manual cropping before model prediction.

### 2.2. Dataset

For the third molar localization task, 737 panoramic radiographs and 2736 annotations were used. The classes were defined as teeth 18, 28, 38, and 48. The images were divided into training, validation, and test subsets at an 80:10:10 ratio. The training set included 590 images and 2179 annotations. The validation set included 78 images and 290 annotations. The test set included 69 images and 267 annotations.

For the developmental stage classification task, 695 panoramic radiographs and 2533 annotations were used. The classes were defined as AB, CD, EF, and GH. The same 80:10:10 split was applied. The training set included 556 images and 2033 annotations. The validation set included 72 images and 265 annotations. The test set included 65 images and 235 annotations.

The panoramic radiographs were retrospectively obtained from the archive of Yozgat Bozok University Faculty of Dentistry. All images were de-identified before annotation and model training. The study protocol was approved by Yozgat Bozok University Ethics Committee with approval number 2025-GOKAEK-254_2025.02.19_317. All procedures are reported in accordance with the Declaration of Helsinki and institutional data protection requirements.

For the supplementary 18-year threshold segmentation analysis, the same radiographic archive was reorganized into two classes: 18 years of age and older and under 18 years of age. This analysis used 695 panoramic radiographs and 2573 labels. The dataset was divided into training, validation, and test subsets at an 80:10:10 ratio, corresponding to 574 training images, 66 validation images, and 55 test images. The training set contained 2129 labels, the validation set contained 240 labels, and the test set contained 204 labels. Class 0 represented the 18-years-and-older group, whereas Class 1 represented the under-18 group. The distribution showed a marked class imbalance, with fewer labels in the 18-years-and-older group (Table 1).

The primary split unit was the panoramic radiograph. All annotations belonging to the same panoramic radiograph were retained within the same subset. Because the images were de-identified before model training, the manuscript does not claim verification of repeated patient identifiers across different radiographs. This point is acknowledged as a limitation and should be addressed by explicit patient-level splitting in future multi-center validation studies.

### 2.3. Model Training

YOLO-based segmentation models were used in both stages. The third molar localization task was performed with yolov8x-seg. For developmental stage classification, three models were compared: yolov8x-seg, yolo11x-seg, and yolo26x-seg. All models were trained using an image size of 1280 pixels. The AdamW optimizer was used, and the learning rate was set to 0.001. Training was planned with an upper limit of 150 epochs, and the best weights were obtained at different epochs due to early stopping.

For the localization model, the best weights were obtained at epoch 99, and training stopped at epoch 124. For developmental stage classification, the best weights were obtained at epoch 37 for yolov8x-seg, epoch 56 for yolo11x-seg, and epoch 98 for yolo26x-seg. Training durations were 3 h 06 min for the localization model, 1 h 30 min for yolov8x-seg, 1 h 58 min for yolo11x-seg, and 4 h 13 min for yolo26x-seg. Model training was performed using an Intel Xeon Gold 5218 CPU, Tesla V100-PCIE-16GB GPU, and 220 GB RAM.

The supplementary binary 18-year threshold model was trained as a YOLO instance segmentation task using yolov8x-seg. The image size was 640 pixels, the planned training length was 800 epochs, and the optimizer and learning rate were recorded as AdamW and 0.001 in the analysis summary. The training configuration also included batch size 8, deterministic training with seed 0, patience 75, IoU threshold 0.7, maximum detections 300, weight decay 0.0005, warm-up epochs 3.0, and the standard Ultralytics augmentation settings recorded in the training configuration, including translation, scale, HSV adjustment, mosaic, auto-augmentation, and random erasing. No additional class weighting, oversampling, or resampling was applied. Therefore, the effect of class imbalance was considered during interpretation rather than being fully corrected during model training (Table 2).

### 2.4. Evaluation Metrics

Model performance was evaluated using true positives (TP), false positives (FP), false negatives (FN), overall accuracy, mAP@0.5, mAP@0.5:0.95, Dice coefficient, and Jaccard index. Precision–recall curves, F1–confidence curves, precision–confidence curves, recall–confidence curves, ROC curves, and confusion matrices were also examined. For visual evaluation, ground-truth labels and model predictions from the test set were compared.

For YOLO instance segmentation, TP, FP, and FN are defined at the object/mask detection level. A true negative count was not calculated because the number of background regions without an object is not finite and is not clinically meaningful in instance segmentation. Therefore, TN-dependent metrics such as specificity and negative predictive value were not reported for the instance segmentation outputs. Recall was interpreted as sensitivity, and precision was interpreted as positive predictive value. For the supplementary binary age threshold analysis, class-wise precision, recall, F1-score, Dice, and Jaccard values were reported together with overall accuracy and mAP values.

**Figure 2:**
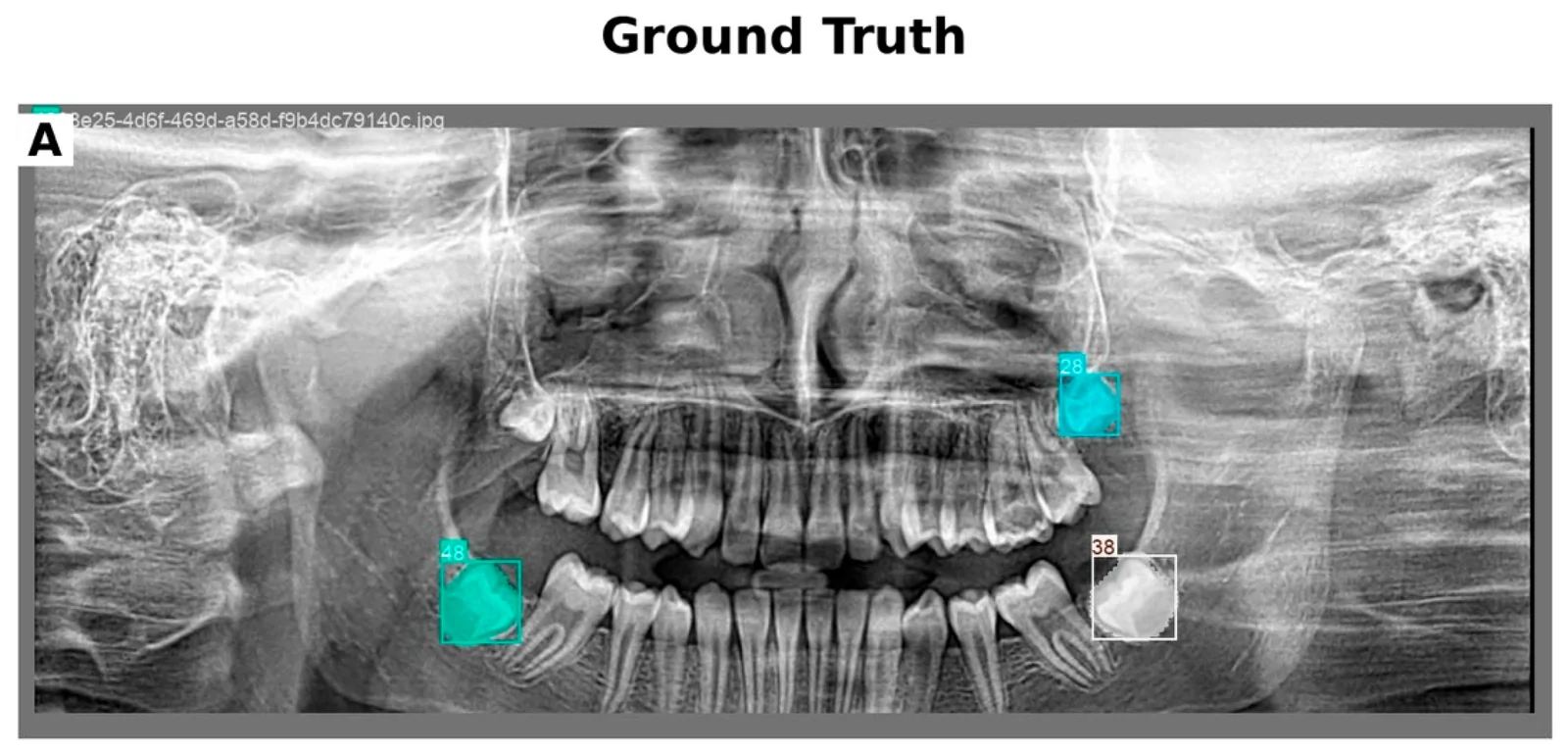

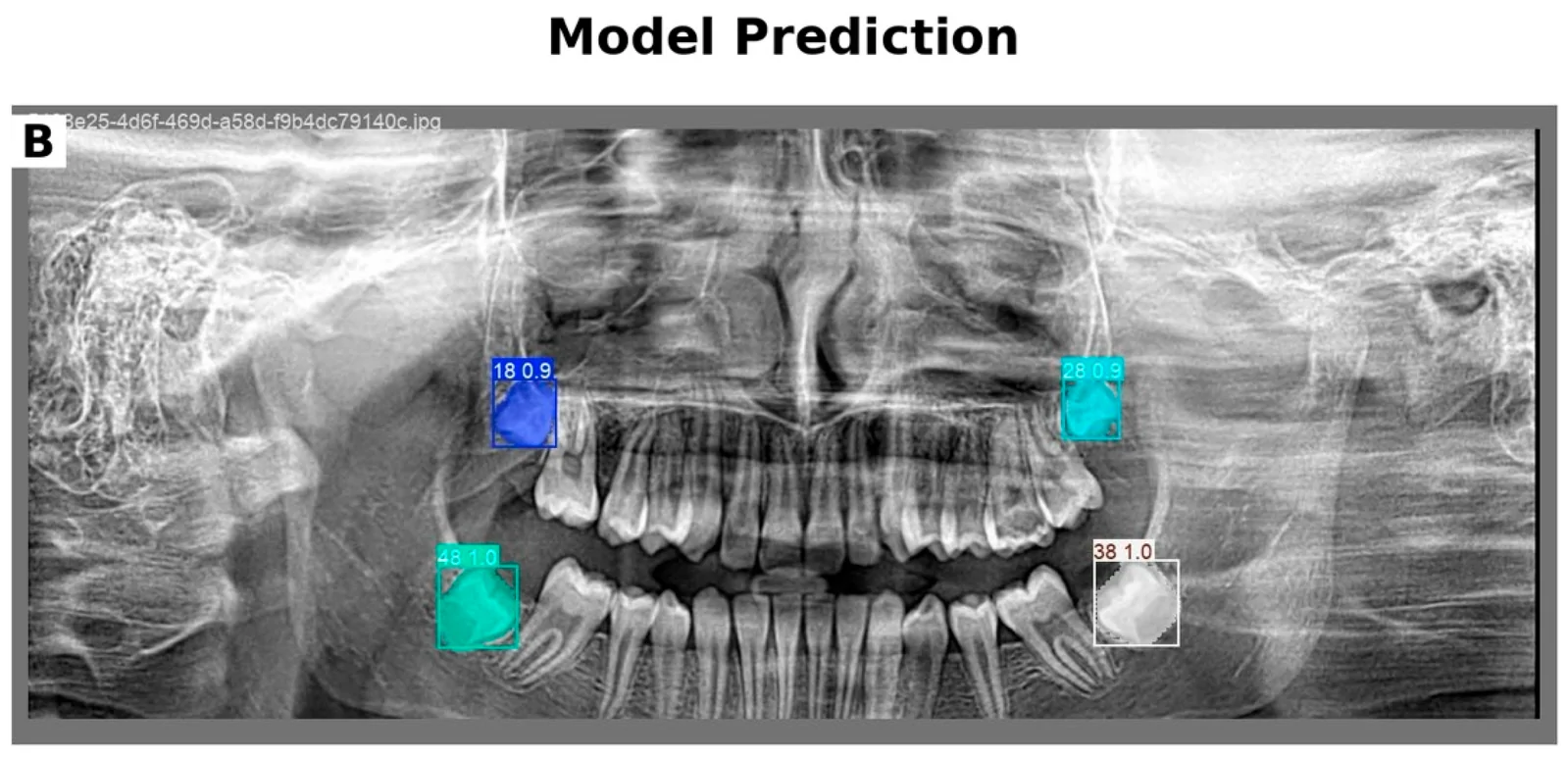
Representative third molar localization outputs: (**A**) ground-truth annotations; (**B**) corresponding model predictions for teeth 18, 28, 38, and 48.

**Figure 3:**
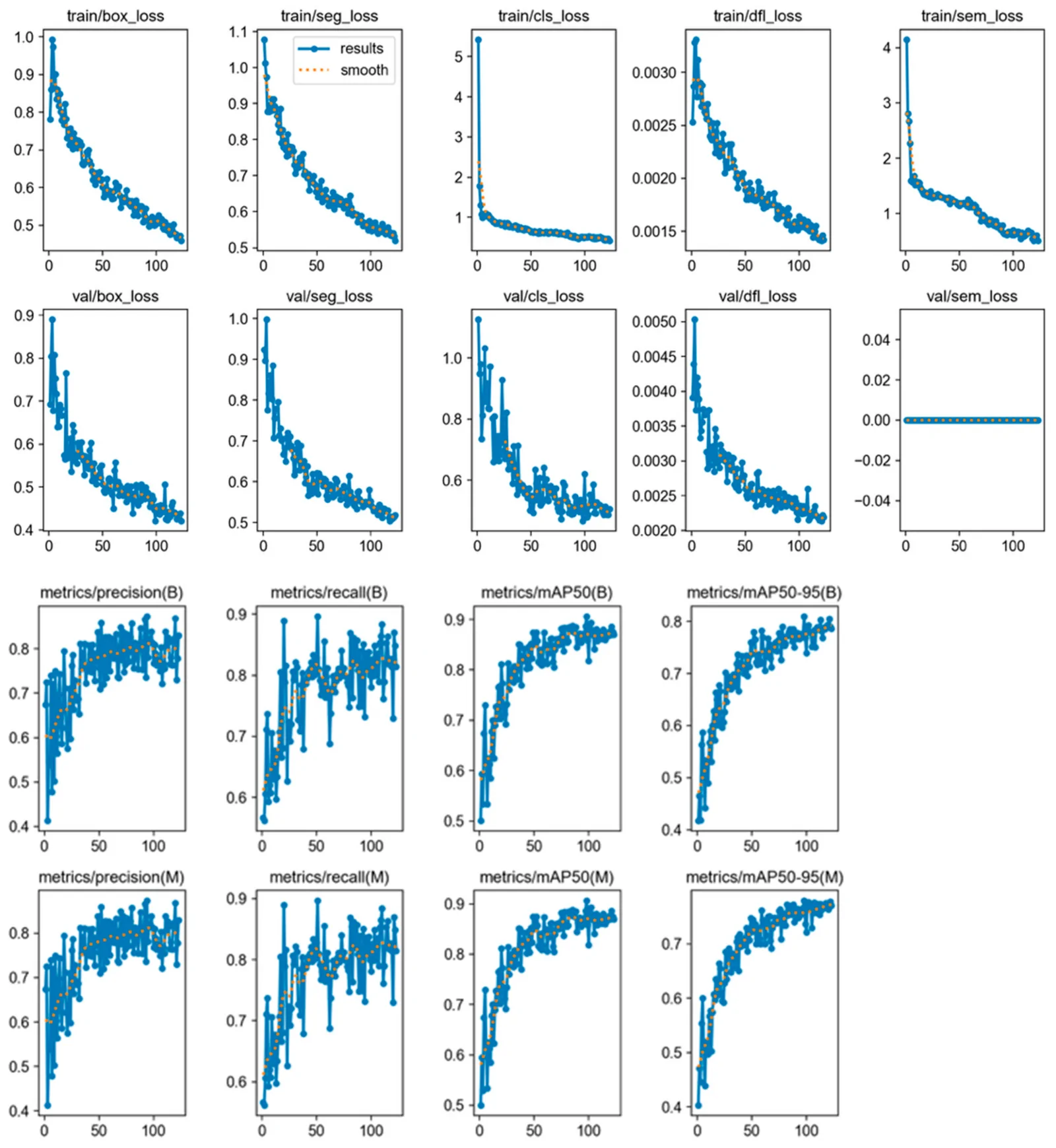
Training and validation curves of the developmental stage segmentation model.

Representative third molar localization outputs are shown in Figure 2, and the corresponding training and validation curves are presented in Figure 3.

## 3. Results

### 3.1. Third Molar Localization Performance

The model used for third molar localization showed high performance in detecting teeth 18, 28, 38, and 48. In the test set, TP = 256, FP = 4, and FN = 11 were obtained. The overall accuracy was 0.9446. The mAP@0.5 value was 0.980, and the mAP@0.5:0.95 value was 0.875. The overall Dice coefficient was 0.9064, and the overall Jaccard index was 0.8696.

Class-wise overlap metrics were also high. The highest Dice coefficient was obtained for tooth 28, and the highest Jaccard index was obtained for tooth 38. These findings indicate that the model was able to localize third molars consistently across maxillary and mandibular regions (Table 3).

To further assess the consistency of segmentation performance across individual third molars, class-wise Dice and Jaccard values were calculated for teeth 18, 28, 38, and 48 (Table 4).

### 3.2. Overall Performance of Developmental Stage Classification Models

The developmental stage classification task was more difficult than third molar localization. Three segmentation models were compared for AB, CD, EF, and GH classification.

The yolov8x-seg model produced TP = 196, FP = 50, and FN = 39. The overall accuracy was 0.6877. The mAP@0.5 value was 0.737, and the mAP@0.5:0.95 value was 0.644. The overall Dice coefficient was 0.5188, and the overall Jaccard index was 0.4962.

The yolo11x-seg model produced TP = 200, FP = 40, and FN = 35. The overall accuracy was 0.7272, mAP@0.5 was 0.762, and mAP@0.5:0.95 was 0.650. The overall Dice coefficient was 0.5783, and the overall Jaccard index was 0.5529. This model provided the most balanced result in terms of accuracy, FP control, Dice, and Jaccard values.

The yolo26x-seg model produced TP = 189, FP = 90, and FN = 46. The overall accuracy was 0.5815. However, this model achieved the highest mAP values, with mAP@0.5 of 0.783 and mAP@0.5:0.95 of 0.697. The higher FP count and lower overlap metrics indicate that this model tended to produce more detections in some stage classes (Table 5).

### 3.3. Class-Wise Dice and Jaccard Results

Class-wise evaluation showed that CD was the most consistently learned developmental stage. For CD, the Dice coefficient was 0.7324 in yolov8x-seg, 0.7691 in yolo11x-seg, and 0.7563 in yolo26x-seg. The corresponding Jaccard values were also higher than those of other classes.

EF showed lower overlap performance across all models. For EF, Dice values were 0.3875 in yolov8x-seg, 0.4353 in yolo11x-seg, and 0.3090 in yolo26x-seg. This finding suggests that EF has more overlap with adjacent developmental groups and is more difficult to separate radiographically (Table 6).

### 3.4. Additional Test-Validation Outputs of yolo26x-seg

The yolo26x-seg model was further examined using detailed test–validation outputs. The overall mAP@0.5 value was 0.783. The highest class-wise AP was obtained for CD, with a value of 0.957. GH, AB, and EF followed CD in descending order. The precision–recall outputs showed that the model separated CD more effectively than the other developmental stage groups.

The ROC analysis showed that the highest AUC was obtained for AB, with a value of 0.6936. AUC values were 0.6546 for EF, 0.6347 for CD, and 0.5911 for GH. All AUC values were above 0.5, but the values remained moderate. This indicates that the model performed above random classification but still had difficulty separating some adjacent developmental stages.

The normalized confusion matrix showed correct classification rates of 0.71 for AB, 0.89 for CD, 0.65 for EF, and 0.71 for GH. CD had the highest correct classification rate, while EF had the lowest. The best mask F1-score was 0.73 at a confidence threshold of 0.420. The highest precision was 1.00 at a confidence threshold of 0.988, while maximum recall was 0.96 at a confidence threshold of 0.000.

**Figure 4:**
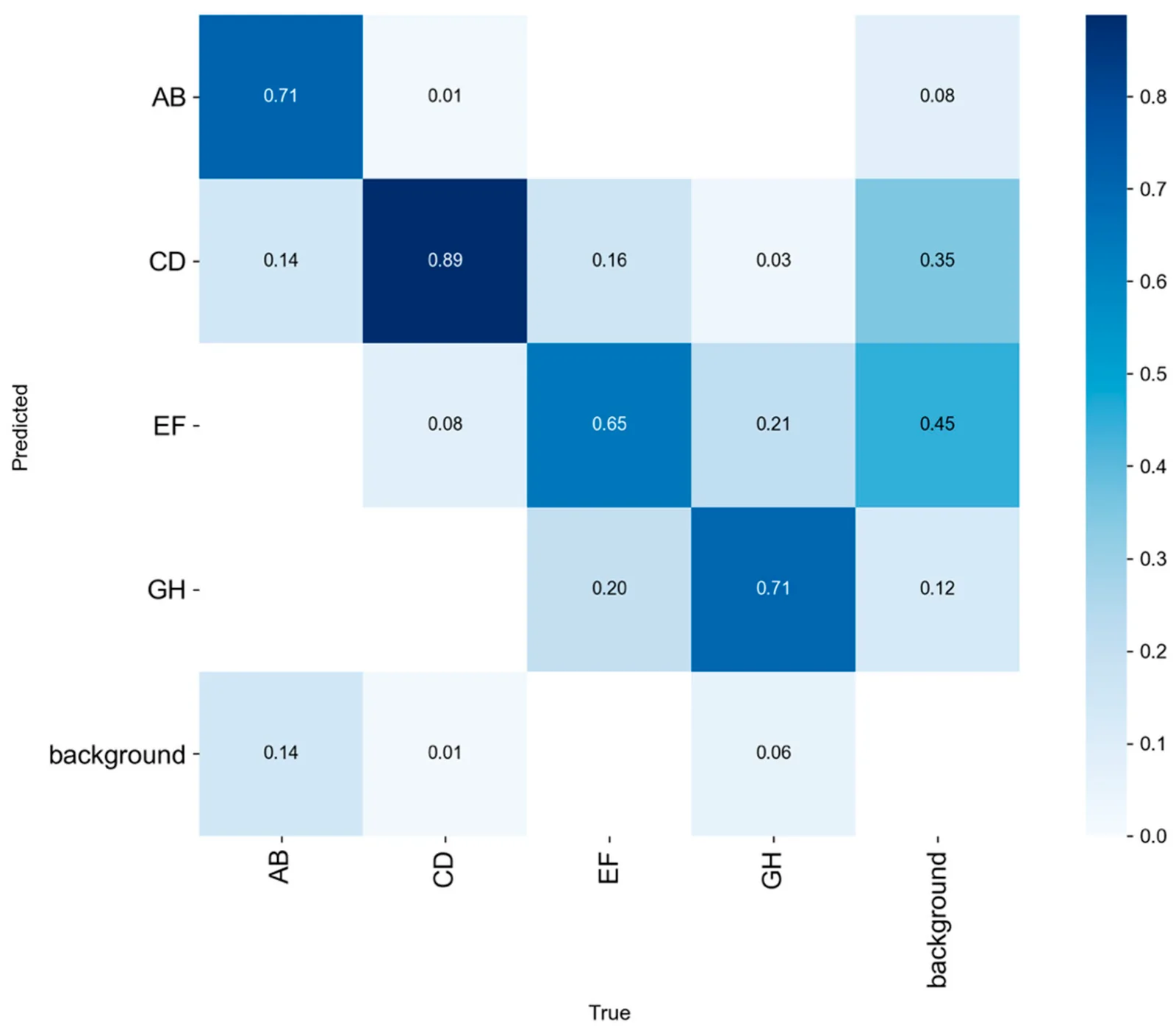
Raw and normalized confusion matrices of the yolo26x-seg model for AB–CD–EF–GH developmental stage classification.

**Figure 5:**
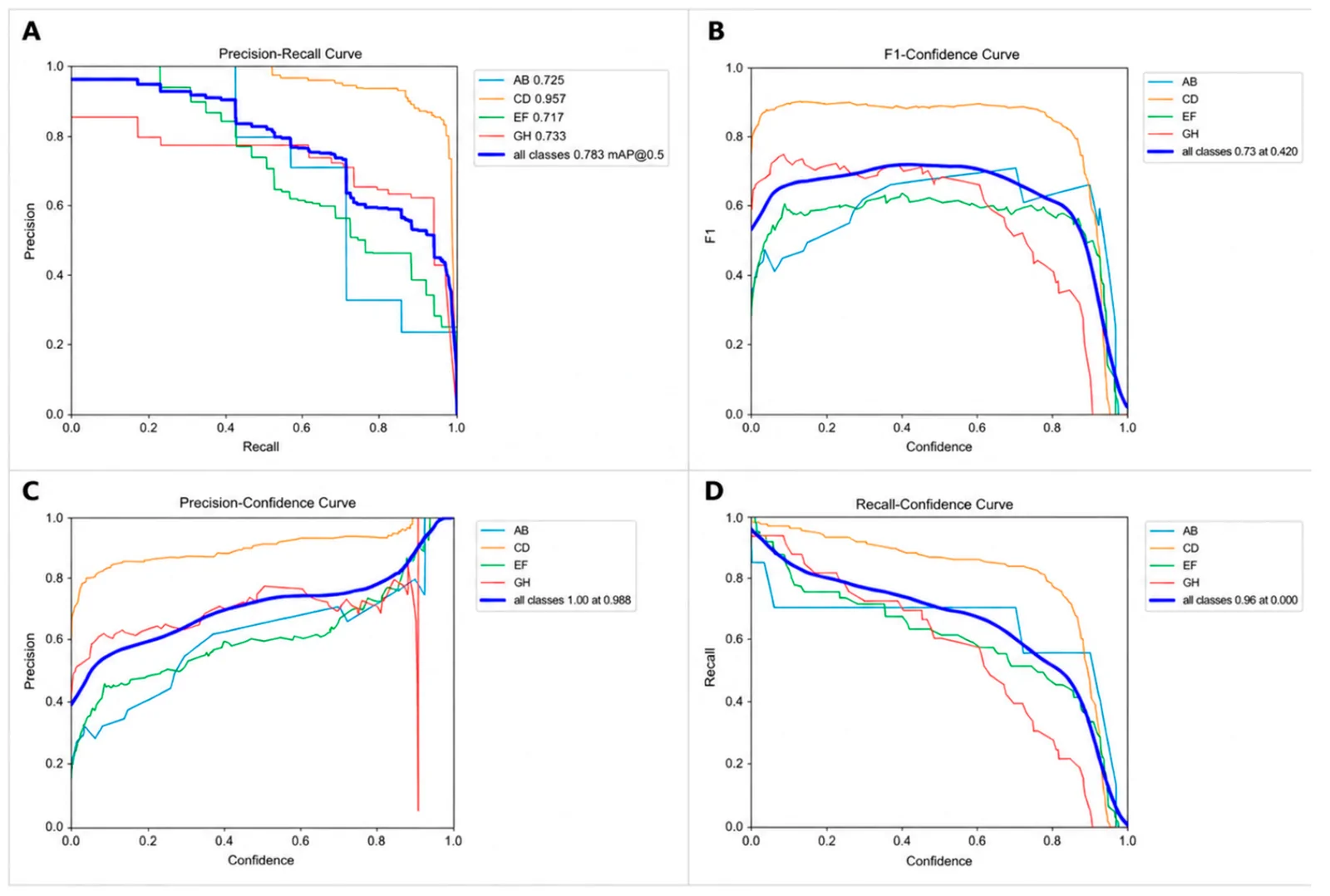
Test-validation performance curves of the yolo26x-seg model: (**A**) precision-recall curve; (**B**) F1-confidence curve; (**C**) precision-confidence curve; (**D**) recall-confidence curve.

Detailed test-validation performance is summarized in Table 7; the corresponding raw and normalized confusion matrices are shown in Figure 4, and the performance curves are presented in Figure 5.

The normalized confusion matrix provides a class-wise view of the model’s prediction distribution and shows where stage-level misclassifications occurred. To further evaluate the behavior of the model across different confidence thresholds, precision–recall, F1–confidence, precision–confidence, and recall–confidence curves were also examined (Figure 5).

### 3.5. Visual Evaluation

Visual outputs showed that third molar regions were generally marked in the correct anatomical areas. In the localization task, teeth 18, 28, 38, and 48 were detected with high consistency. In the developmental stage task, CD predictions appeared more stable, while EF and GH predictions showed more class overlap in some samples.

**Figure 6:**
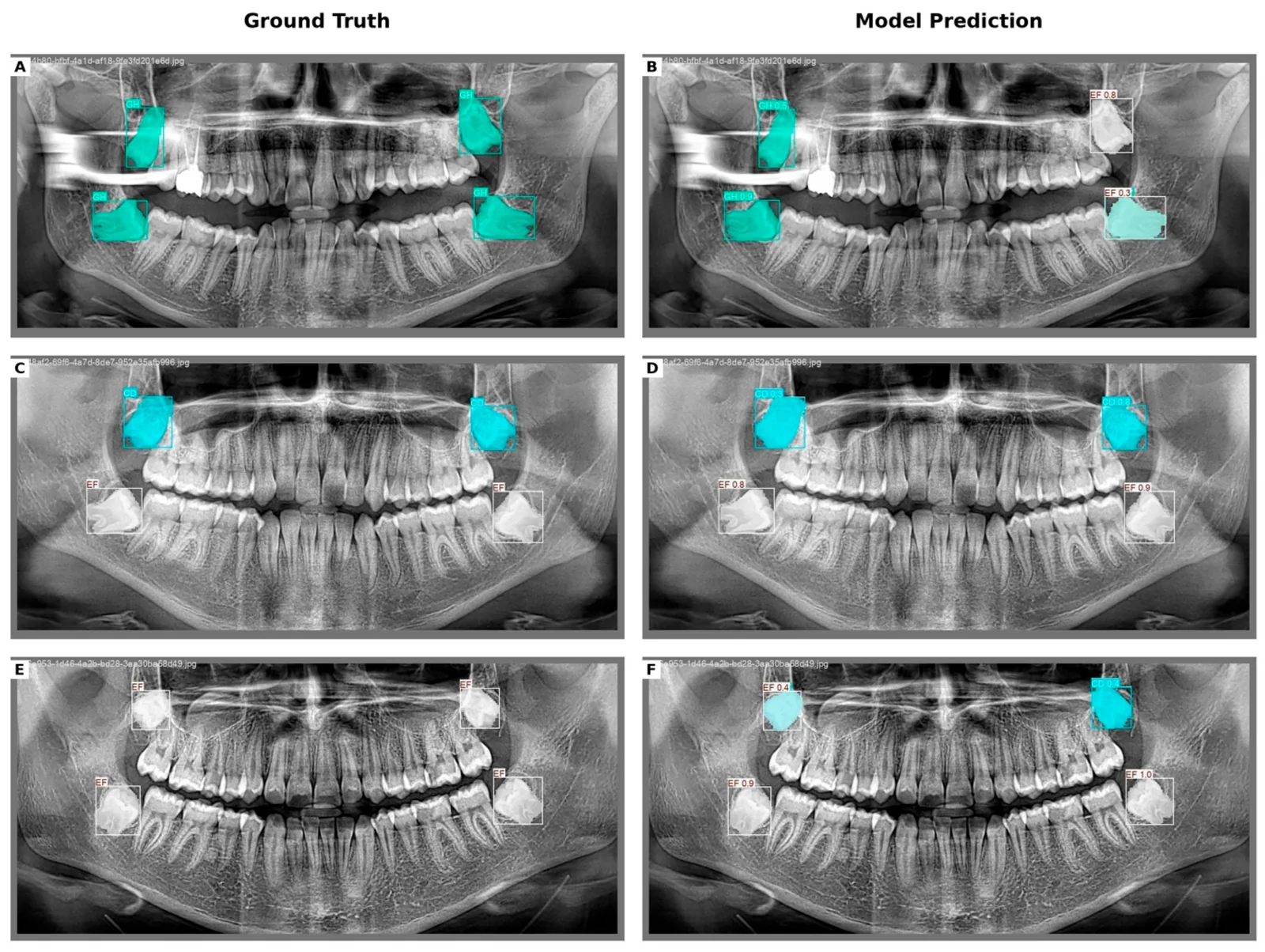
Representative ground-truth and prediction examples for developmental stage segmentation using the yolo26x-seg model: (**A**,**C**,**E**) ground-truth annotations; (**B**,**D**,**F**) corresponding model predictions.

Comparison of ground-truth and prediction images supported the numerical findings. CD stage segmentations were more consistent, whereas EF and GH stage boundaries were more likely to overlap in transitional cases (Figure 6).

### 3.6. Supplementary 18-Year Threshold Segmentation Analysis

A supplementary binary segmentation analysis was performed to evaluate whether the model output could be related to the legally relevant 18-year threshold. The classes were defined as 18 years of age and older (Class 0) and under 18 years of age (Class 1). This analysis used 695 panoramic radiographs and 2573 labels, divided into 574 training images, 66 validation images, and 55 test images. The test set contained 204 labels.

The binary 18-year threshold model produced TP = 172, FP = 37, and FN = 32. The overall accuracy was 0.8152, mAP@0.5 was 0.635, and mAP@0.5:0.95 was 0.563. Based on TP, FP, and FN, the overall precision was 0.8230, recall was 0.8431, and F1-score was 0.8329. Because this was an instance segmentation evaluation, TN was not defined and specificity and NPV were not calculated (Table 8).

Class-wise analysis showed an asymmetric performance profile. For the 18-years-and-older class, precision was 0.6154, recall was 0.2353, and F1-score was 0.3404. The corresponding Dice and Jaccard values were 0.1996 and 0.1885. For the under-18 class, precision was 0.8367, recall was 0.9647, and F1-score was 0.8962. The corresponding Dice and Jaccard values were 0.7592 and 0.7282. These findings indicate that the binary model learned the under-18 class more consistently than the 18-years-and-older class (Table 9).

## 4. Discussion

This study evaluated third molar localization and Demirjian-based developmental stage classification on panoramic radiographs using YOLO-based segmentation models. The findings should be read through two linked tasks. The first task was anatomical localization of teeth 18, 28, 38, and 48. The second task was stage classification into AB, CD, EF, and GH groups. The difference between these tasks was clear. Third molar localization behaved as a high-performance detection problem, whereas developmental staging required interpretation of gradual crown–root morphology, root length, canal wall configuration, and apical maturation.

The localization model performed strongly on the test set, with TP = 256, FP = 4, and FN = 11. The overall accuracy of 0.9446, mAP@0.5 of 0.980, and mAP@0.5:0.95 of 0.875 indicate that third molars can be identified with high reliability on panoramic radiographs. The Dice coefficient of 0.9064 and Jaccard index of 0.8696 also show that the model achieved strong spatial agreement with the annotated regions. The highest Dice value was obtained for tooth 28, and the highest Jaccard value was obtained for tooth 38. These findings suggest that the model learned maxillary and mandibular third molar positions despite differences in angulation, impaction pattern, and surrounding anatomical overlap.

This localization result is consistent with the broader literature on tooth detection and segmentation. Peker and Kurtoglu reported precision of 0.90, recall of 0.94, mAP50 of 0.968, and F1-score of 0.919 for YOLOv10-based tooth detection and numbering in pediatric panoramic radiographs [28]. Putra et al. reported 88.5% accuracy, 87.70% precision, 100% recall, and 93.44% F1-score using a YOLOv4-based approach for automated permanent tooth detection and numbering [30]. Leite et al. found that an AI-driven tool detected and segmented teeth on panoramic radiographs faster than manual segmentation and with high accuracy [31]. Bilgir et al. also reported successful automated tooth detection and numbering on panoramic radiographs using a deep convolutional neural network [32]. These findings support the present observation that tooth-level localization can be performed more reliably than developmental staging when the target object has a recognizable anatomical boundary.

Developmental stage classification was more challenging. The yolo11x-seg model produced the most balanced profile, with TP = 200, FP = 40, FN = 35, overall accuracy of 0.7272, Dice coefficient of 0.5783, and Jaccard index of 0.5529. The yolo26x-seg model achieved the highest mAP@0.5 and mAP@0.5:0.95 values, at 0.783 and 0.697, respectively, but produced more FP detections and a lower overall accuracy of 0.5815. This pattern is important because mAP and overlap metrics do not describe the same behavior. mAP rewards detection performance across thresholds and intersection-over-union levels, while Dice and Jaccard measure the spatial agreement between the predicted mask and the reference annotation. A model may therefore achieve higher mAP while still producing less stable masks or additional detections.

The findings on model selection are in line with recent dental AI studies showing that architectural scale alone does not guarantee clinically preferable behavior. Beşer et al. evaluated YOLOv5 for tooth detection, segmentation, and numbering in mixed dentition and emphasized the usefulness of object-level outputs in panoramic radiographs [29]. Peker and Kurtoglu reported strong tooth detection metrics with YOLOv10 in a pediatric population but also underlined the need for optimization across varied clinical cases [28]. In a third-molar-specific context, Akdoğan et al. used YOLO11 submodels for assessing mandibular third molar extraction difficulty and reported precision of 97.00%, recall of 94.55%, and F1-score of 95.76% for their developed pipeline [34]. Chindanuruks et al. trained a CNN-based model for impacted mandibular third molar detection and surgical difficulty classification using 1730 panoramic radiographs [35]. These studies show that YOLO and CNN-based methods can perform well in dental tasks, but their clinical usefulness depends on the balance between detection, false-positive control, segmentation overlap, and interpretability.

Class-wise results provide further insight into third molar developmental morphology. CD was the most stable developmental group across all models. The Dice coefficient for CD was 0.7324 with yolov8x-seg, 0.7691 with yolo11x-seg, and 0.7563 with yolo26x-seg. In the yolo26x-seg outputs, the AP value for CD was 0.957 and the normalized correct classification rate was 0.89. CD represents a stage range in which crown morphology is largely established and initial root development becomes visible. These features provide clearer radiographic cues than early crown mineralization or late apical closure, which may explain why the model learned this group more consistently.

EF had the weakest overlap performance across models. The EF Dice values were 0.3875 for yolov8x-seg, 0.4353 for yolo11x-seg, and 0.3090 for yolo26x-seg. In the yolo26x-seg outputs, EF had an AP value of 0.717 and a normalized correct classification rate of 0.65. EF corresponds to a transitional period in which root formation is ongoing, but the apical region remains open. Root length, canal wall parallelism, apical morphology, and projection quality can vary substantially at this stage. Kayaci et al. reported a similar difficulty in root development classification of permanent first molars, where detection performance was high but stage-level accuracy varied across developmental subsets [37]. This supports the present interpretation that root development staging is intrinsically more difficult than tooth localization. Earlier CNN-based studies also demonstrated the feasibility of automated tooth segmentation and numbering on panoramic radiographs [38,39], reinforcing this contrast between anatomical localization and developmental staging.

GH also requires cautious interpretation. In yolo26x-seg, the AP value for GH was 0.733, the normalized correct classification rate was 0.71, and the AUC value was 0.5911. GH represents late root maturation and apical closure. Although this group may appear advanced from a clinical viewpoint, panoramic radiographs may show the apical region with superimposition, mandibular canal proximity, root curvature, or projection-related blur. These factors can lead to confusion between EF and GH, especially when apical closure is partial or not sharply visible. Third molar impaction adds another source of variability, as shown in AI studies on extraction difficulty and impaction classification [34,35,36].

AB had a normalized correct classification rate of 0.71 in the yolo26x-seg outputs. This result should be interpreted together with the class distribution. AB was represented by fewer annotations than CD, EF, and GH. In smaller classes, the model has fewer examples of variation in shape, size, contrast, and position. AB also includes early mineralization patterns with limited visible morphological markers. Increasing the number of AB samples, using balanced sampling, applying class weighting, and using targeted augmentation may improve the stability of this group.

The decision to group Demirjian stages as AB, CD, EF, and GH provided a practical structure for model training. The original Demirjian method includes eight stages from A to H [1,2]. In manual radiographic staging, neighboring stages may be difficult to separate, particularly when crown–root morphology is close to a transition point [3,5,10]. Pairing adjacent stages increases the number of observations in each class and creates broader stage boundaries. Even with this simplified grouping, EF and GH remained difficult to separate. This result shows that third molar development is a continuous biological process, whereas supervised deep learning requires discrete labels.

Third molars remain valuable in forensic age assessment because their development continues later than most other permanent teeth. They are therefore relevant for adolescents and young adults, especially in evaluations near legal age thresholds [6,7,8,9,10,11,12,13,14,40]. At the same time, third molar maturation varies by population background, sex, jaw side, impaction status, agenesis, genetic structure, and imaging quality [6,7,8,9,10,11,12,13]. The primary AB/CD/EF/GH model in this study should be interpreted as a developmental staging model rather than a direct chronological age estimator. To address the forensic relevance of the 18-year threshold more concretely, a supplementary binary segmentation experiment was added with classes defined as 18 years of age and older and under 18 years of age.

The supplementary binary model yielded an overall accuracy of 0.8152, precision of 0.8230, recall of 0.8431, F1-score of 0.8329, mAP@0.5 of 0.635, and mAP@0.5:0.95 of 0.563. These values suggest that the model can provide a preliminary age threshold signal. However, the class-wise results also show that this signal is not yet suitable for stand-alone forensic decision-making. The 18-years-and-older class had low recall (0.2353), low Dice (0.1996), and low Jaccard (0.1885), while the under-18 class had substantially stronger recall (0.9647), F1-score (0.8962), Dice (0.7592), and Jaccard (0.7282). This imbalance is clinically important. Missing individuals who are actually 18 years of age or older would be unacceptable in some legal contexts, whereas over-calling adulthood may be unacceptable in others. The model should therefore be used only as an interpretable auxiliary output until validated on larger, balanced, patient-level, external datasets.

The additional test–validation outputs of yolo26x-seg show how confidence threshold selection affects model behavior. The best mask F1-score was 0.73 at a confidence threshold of 0.420, indicating that the best balance between precision and recall occurred at a moderate confidence level. Precision reached 1.00 at a confidence threshold of 0.988, but such a strict threshold may miss true findings. Maximum recall was 0.96 at a confidence threshold of 0.000, which captures more true annotations but increases FP detections. This behavior is clinically meaningful. A screening workflow may tolerate lower thresholds to reduce missed cases, whereas a reporting workflow may require higher precision.

The ROC outputs were retained as numerical AUC values, but the visual interpretation of model behavior was based mainly on precision–recall and confidence curves. In yolo26x-seg, AUC values were 0.6936 for AB, 0.6347 for CD, 0.6546 for EF, and 0.5911 for GH. These values were above chance but remained moderate. In imbalanced or multi-class detection settings, ROC curves can be less informative than precision–recall curves because precision–recall plots directly reflect the balance between positive detections and missed targets [41]. For this reason, the main performance figure was revised to focus on precision–recall, F1–confidence, precision–confidence, and recall–confidence curves.

One strength of this study is the two-stage analytical structure. Localizing the third molar first makes the developmental stage task more anatomically controlled. Whole-image age prediction models may produce a numerical result without showing which dental structures influenced the decision. In contrast, segmentation-based outputs show the tooth region and the predicted stage together. This visible intermediate output is relevant for forensic dentistry, where a model result should be open to visual checking and expert review.

Another strength is the comparison of three YOLO-based models under similar training conditions. The comparison showed that a newer or larger model did not automatically provide the most balanced result. yolo26x-seg produced the highest mAP values, whereas yolo11x-seg provided better overall accuracy, lower FP count, and higher Dice and Jaccard values. This finding supports a multi-metric evaluation strategy. In dental AI studies, performance should not be judged by a single summary metric. Class-specific errors, segmentation overlap, threshold behavior, and the clinical purpose of the model should be evaluated together.

Recent medical AI research also points to future methodological directions that are relevant to this task. Knowledge distillation and teacher–student learning may help transfer information from high-capacity segmentation models to lighter models that can be deployed more efficiently in clinical or forensic workflows [42]. Personalized federated learning approaches such as PHH-FL may be useful when multi-center dental radiographic datasets cannot be pooled directly because of institutional or privacy restrictions [43]. Attention-based multi-modal architectures, although developed in other sensing domains, also emphasize the potential value of combining local structural cues with global contextual information [44]. These approaches were not implemented in the present study, but they may help future third molar models address small datasets, class imbalance, and external generalizability.

This study also has limitations. The dataset was divided into training, validation, and test subsets, which provides an internal evaluation structure. All annotations belonging to the same panoramic radiograph were kept within the same subset; however, explicit repeated-patient verification across different radiographs was not available after de-identification. This prevents a stronger claim of fully verified patient-level separation. External validation using radiographs from different institutions, devices, age distributions, and population backgrounds is still required. Dental age estimation and third molar development are population-sensitive, and performance values obtained from one dataset should not be transferred directly to another population [20,21,22,23]. Future studies should report sex distribution, age distribution, impaction status, jaw side, imaging device, image quality, annotation agreement, and patient-level split verification.

Another limitation is that the developmental stage model was not converted into a full chronological age prediction model. The supplementary 18-year threshold segmentation analysis provides an age-threshold experiment, but it does not replace formal forensic age estimation. The present outputs should be interpreted as segmentation-based developmental or threshold support outputs. Future work should link AB/CD/EF/GH stage probabilities to chronological age distributions, sex-specific models, population-specific thresholds, calibration curves, and case-level estimates of sensitivity, specificity, positive predictive value, negative predictive value, and uncertainty intervals. Such analyses are particularly important for legal thresholds such as 18 years [6,7,14,40].

Reporting standards should also be considered in future versions of this work. The CLAIM checklist recommends detailed reporting of dataset sources, reference standards, model development, validation, performance metrics, and clinical applicability in AI-based medical imaging studies [45]. The TRIPOD + AI statement similarly emphasizes transparent reporting of data sources, participant selection, model development, validation, and performance evaluation in machine learning prediction models [46]. In response, the revised manuscript provides additional details on model data flow, inference steps, class imbalance, preprocessing, hyperparameters, threshold analysis, code availability, and data availability. Future work should add external validation, patient-level resplitting where repeated radiographs exist, calibration analysis, ablation experiments, and comparison with non-YOLO architectures.

In summary, third molar localization was highly successful, while developmental stage classification remained more sensitive to class morphology and model choice. CD was the most consistently learned group, whereas EF and GH showed more overlap and stage-level confusion. The supplementary 18-year threshold segmentation model provided clinically relevant threshold information but showed unequal class-wise performance, particularly for the 18-years-and-older class. These findings suggest that segmentation-based third molar analysis is feasible as an interpretable support workflow, but its performance should be reported with multiple complementary metrics and validated externally before forensic use.

## 5. Conclusions

This study presents a segmentation-based deep learning framework for third molar localization, Demirjian-based developmental stage classification, and supplementary 18-year threshold analysis on panoramic radiographs. The localization model detected teeth 18, 28, 38, and 48 with high performance, achieving an overall accuracy of 0.9446, mAP@0.5 of 0.980, Dice coefficient of 0.9064, and Jaccard index of 0.8696. Developmental stage classification was more difficult. Among the evaluated stage models, yolo11x-seg provided the most balanced overall profile, while yolo26x-seg achieved the highest mAP values but produced more false positives. The supplementary 18-year threshold model achieved an overall accuracy of 0.8152, precision of 0.8230, recall of 0.8431, and F1-score of 0.8329, but the 18-years-and-older class showed low overlap performance.

Class-wise results showed that CD was the most consistently learned developmental group, while EF had lower overlap performance across all models. These findings suggest that the difficulty of automatic third molar staging is mainly related to transitional morphology between adjacent developmental stages. Segmentation-based staging and binary age threshold analysis offer interpretable structures because the detected tooth region and predicted class can be visually inspected. However, these outputs should not be used as stand-alone legal age decisions. Future studies should use larger, balanced, multi-center datasets, explicit patient-level splits, external validation, calibration, ablation experiments, and non-YOLO baseline comparisons before forensic implementation.

## Figures and Tables

**Table 1:** (**A**) Dataset distribution. (**B**) Distribution of labels in the supplementary 18-year threshold segmentation analysis.

(**A**)				
**Dataset Property**	**Third Molar Localization**		**Developmental Stage Classification**	
Analysis type	Third molar localization		Developmental stage classification	
Classes	18, 28, 38, 48		AB, CD, EF, GH	
Total radiographs	737		695	
Total annotations	2736		2533	
Training images	590		556	
Validation images	78		72	
Test images	69		65	
Training annotations	2179		2033	
Validation annotations	290		265	
Test annotations	267		235	
(**B**)				
**Class**	**Training Labels**	**Validation Labels**	**Test Labels**	**Total Labels**
18 years of age and older (Class 0)	278	38	34	350
Under 18 years of age (Class 1)	1851	202	170	2223
Total	2129	240	204	2573

**Table 2:** Model training characteristics.

Model Training Property	Localization/yolov8x-seg	Stage/yolov8x-seg	Stage/yolo11x-seg	Stage/yolo26x-seg
Analysis type	Localization	Developmental stage	Developmental stage	Developmental stage
Model	yolov8x-seg	yolov8x-seg	yolo11x-seg	yolo26x-seg
Image size	1280	1280	1280	1280
Epoch upper limit	150	150	150	150
Best weights	99th epoch	37th epoch	56th epoch	98th epoch
Training stopped	124th epoch	62nd epoch	81st epoch	123rd epoch
Optimizer	AdamW	AdamW	AdamW	AdamW
Learning rate	0.001	0.001	0.001	0.001
Training time	3 h 06 min	1 h 30 min	1 h 58 min	4 h 13 min
Single-image prediction time	57.2 ms	57.6 ms	66.4 ms	66.8 ms

**Table 3:** Overall performance of the third molar localization model.

Performance Metric	Value
Model	yolov8x-seg
Classes	18, 28, 38, 48
Test images	69
Test annotations	267
TP	256
FP	4
FN	11
Overall accuracy	0.9446
mAP@0.5	0.980
mAP@0.5:0.95	0.875
Overall Dice	0.9064
Overall Jaccard	0.8696

**Table 4:** Class-wise Dice and Jaccard values for third molar localization.

Tooth Number	Mean Dice	Mean Jaccard
18	0.8779	0.8397
28	0.9339	0.8894
38	0.9218	0.8902
48	0.8920	0.8592
Overall	0.9064	0.8696

**Table 5:** Overall performance comparison of developmental stage classification models.

Performance Metric	yolov8x-seg	yolo11x-seg	yolo26x-seg
Test images	65	65	65
Test annotations	235	235	235
TP	196	200	189
FP	50	40	90
FN	39	35	46
Overall accuracy	0.6877	0.7272	0.5815
mAP@0.5	0.737	0.762	0.783
mAP@0.5:0.95	0.644	0.650	0.697
Overall Dice	0.5188	0.5783	0.4730
Overall Jaccard	0.4962	0.5529	0.4544

**Table 6:** Class-wise Dice and Jaccard values of developmental stage classification models.

Performance Metric	yolov8x-seg	yolo11x-seg	yolo26x-seg
AB Dice	0.4703	0.5281	0.3324
AB Jaccard	0.4444	0.4983	0.3168
CD Dice	0.7324	0.7691	0.7563
CD Jaccard	0.7016	0.7372	0.7261
EF Dice	0.3875	0.4353	0.3090
EF Jaccard	0.3721	0.4173	0.2976
GH Dice	0.4848	0.5805	0.4941
GH Jaccard	0.4665	0.5588	0.4771
Overall Dice	0.5188	0.5783	0.4730
Overall Jaccard	0.4962	0.5529	0.4544

**Table 7:** Detailed test–validation performance summary of yolo26x-seg.

Metric	Value
Overall mAP@0.5	0.783
AP for AB	0.725
AP for CD	0.957
AP for EF	0.717
AP for GH	0.733
Best mask F1	0.73 at confidence 0.420
Precision peak	1.00 at confidence 0.988
Maximum recall	0.96 at confidence 0.000
AUC for AB	0.6936
AUC for CD	0.6347
AUC for EF	0.6546
AUC for GH	0.5911
Normalized correct classification for AB	0.71
Normalized correct classification for CD	0.89
Normalized correct classification for EF	0.65
Normalized correct classification for GH	0.71

**Table 8:** Overall performance of the supplementary 18-year threshold segmentation model.

Metric	Value
Model	yolov8x-seg
Classes	18 years and older/Under 18
Training images	574
Validation images	66
Test images	55
Training labels	2129
Validation labels	240
Test labels	204
TP	172
FP	37
FN	32
Overall accuracy	0.8152
Precision/PPV	0.8230
Recall/sensitivity	0.8431
F1-score	0.8329
mAP@0.5	0.635
mAP@0.5:0.95	0.563
Overall Dice	0.4794
Overall Jaccard	0.4584

**Table 9:** Class-wise performance of the supplementary 18-year threshold segmentation model.

Class	TP	FP	FN	Precision	Recall/Sensitivity	F1-Score	Dice	Jaccard
18 years of age and older (Class 0)	8	5	26	0.6154	0.2353	0.3404	0.1996	0.1885
Under 18 years of age (Class 1)	164	32	6	0.8367	0.9647	0.8962	0.7592	0.7282

## Data Availability

The data presented in this study are available on request from the corresponding author. The data are not publicly available due to privacy and ethical reasons. The model training and evaluation scripts are available from the corresponding author upon reasonable request, subject to institutional approval and restrictions related to data access and reproducibility of the local training environment. The model training and evaluation scripts are available from the corresponding author upon reasonable request, subject to institutional approval and restrictions related to data access and reproducibility of the local training environment.

## Abbreviations

AI: artificial intelligence; AP: average precision; AUC: area under the curve; CNN: convolutional neural network; DL: deep learning; FN: false negative; FP: false positive; mAP: mean average precision; NPV: negative predictive value; PPV: positive predictive value; PR: panoramic radiograph; ROC: receiver operating characteristic; ROI: region of interest; TN: true negative; TP: true positive; YOLO: You Only Look Once.
